# *Arcanobacterium canis* strain DSM 25104 isolated from an English bulldog suffering from otitis externa: complete genome sequence

**DOI:** 10.1128/mra.00624-23

**Published:** 2023-12-15

**Authors:** Maria Borowiak, Antonia Kreitlow, Burkhard Malorny, Christoph Lämmler, Ellen Prenger-Berninghoff, Madeleine Plötz, Amir Abdulmawjood

**Affiliations:** 1 Department for Biological Safety, German Federal Institute for Risk Assessment (BfR), Berlin, Germany; 2 Institute of Food Quality and Food Safety, University of Veterinary Medicine Hannover, Hannover, Germany; 3 Institut für Hygiene und Infektionskrankheiten der Tiere, Justus-Liebig-Universität Gießen, Gießen, Germany; University of Maryland School of Medicine, Baltimore, Maryland, USA

**Keywords:** *Arcanobacterium canis*, otitis externa, English bulldog

## Abstract

Many species of the genus *Arcanobacterium* are known as opportunistic pathogens and have been isolated in association with infectious diseases in humans and animals. Here, we present the complete genome sequence of another opportunistic pathogenic representative, namely *Arcanobacterium canis*, isolated from the otitis externa of an English bulldog.

## ANNOUNCEMENT

Gram-positive, facultative anaerobic rods of the genus *Arcanobacterium* occur in both human and animal hosts. The latter includes domestic animals, such as dogs (1, 2), cats (2), and horses (3), livestock, such as cattle (4) or sheep (5), and wildlife animals, such as seals, sea lions, and sea otters (6).

Many species of the genus *Arcanobacterium* show opportunistic pathogenic potential. Examples of *Arcanobacterium* strains linked with infections in animals include *Arcanobacterium bovis* associated with bovine mastitis (4), *Arcanobacterium phocae* isolated from abscesses in marine mammals (6), as well as from skin lesions in mink (7, 8), and *Arcanobacterium pluranimalium* connected with cases of abortions in sheep (5). Here, we announce the complete genome sequence of *Arcanobacterium canis* DSM 25104 (originally named P6775), another potentially pathogenic species of the *Arcanobacterium* genus isolated from a canine otitis externa in 2010 (1, 2).

*Arcanobacterium canis* strain DSM 25104 was retrieved from the cryo-culture collection and cultivated on sheep blood agar for 48  h at 37°C under microaerobic conditions. Genomic DNA was extracted using the PureLink Genomic DNA Mini kit (Thermo Fisher Scientific). To generate a high-quality hybrid assembly, Illumina and Oxford Nanopore Technologies (ONT) sequencing was performed based on the same DNA preparation.

For Illumina short-read data generation, a sequencing library was prepared using the Illumina DNA Prep (M) Tagmentation kit and sequenced in 2 ×  301-bp cycles on an Illumina MiSeq sequencer using the MiSeq reagent kit v3. The short reads were trimmed using fastp v0.19.5 (9), resulting in 0.7 million high-quality paired-end reads (≥81.3% Q30).

For ONT long-read sequencing, a sequencing library was prepared using the SQK-RBK110.96 kit. Sequencing was performed using a FLO-MIN106 flowcell and a MinIon Mk1C device. The generated fast5 data were subsequently basecalled on a GPU server using guppy v6.0.1 (https://nanoporetech.com/community) in SUP mode. The resulting reads in fastq format were trimmed using Porechop v0.2.3 (https://github.com/rrwick/Porechop) and further processed and statistically evaluated using NanoFilt v2.7.1 and NanoStat v1.2.1 (10). In the end, 13,492 reads with a read length *N*_50_ value of 4,319  bp and a mean read quality score of 14.0 were available.

Both data sets were *de novo* assembled and circularized using Unicycler v0.4.4, including pilon (11 – 13). The high-quality hybrid assembly (long-read coverage: 23× and short-read coverage: 100×) consists of one closed chromosome of 1,873,758  bp with a G + C content of 54.5%. Annotation was performed by PGAP v6.5 (14). For all the applied software mentioned above, default parameters were used.

**Fig 1:**
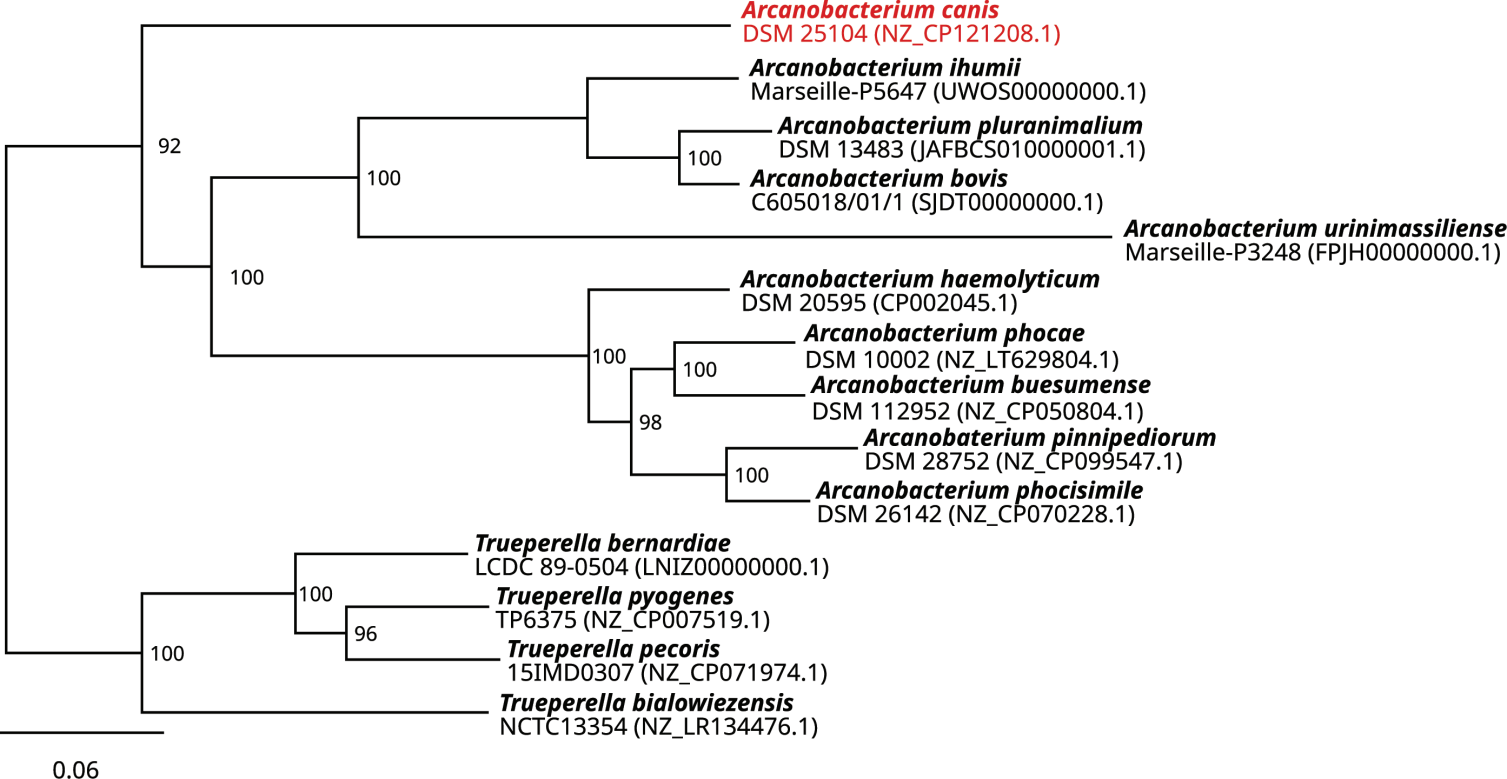
Maximum likelihood phylogenetic tree of the *Arcanobacterium* and the closely related *Truperella* genus generated by comparing the amino acid sequences of 107 core genes using bcgtree. Numbers at branches designate bootstrap support values resulting from 100 bootstrap replicates.

To place the new *A. canis* whole genome sequence in the steadily growing phylogenetic tree of the *Arcanobacterium* genus, a comparison of the amino acid sequence of 107 core genes was conducted using bcgTree v1.1.0 (15). Therefore, *Arcanobacterium* and closely related *Truperella* genome assemblies were downloaded in fasta format from NCBI and uniformly annotated with Prokka 1.14.0 (16). The resulting .faa files were used as input for bcgTree. The generated tree was visualized in Geneious 2020.2.2 and finalized in InkScape v2. The results revealed that *A. canis* forms its own branch in the phylogenetic tree of the *Arcanobacterium* genus (Fig. 1).

## Data Availability

The sequencing read data sets [ONT (SRA: SRX19819636) and Illumina (SRA: SRX19819635)] and the assembly (GenBank: CP121208.1) of *Arcanobacterium canis* have been deposited in the NCBI database under the Bioproject PRJNA950321.
